# Dissecting metabolic syndrome components: data from an epidemiologic survey in a genetic isolate

**DOI:** 10.1186/s40064-015-1049-9

**Published:** 2015-07-07

**Authors:** Ginevra Biino, Maria Pina Concas, Hellas Cena, Debora Parracciani, Simona Vaccargiu, Massimiliano Cosso, Francesca Marras, Vittoria D’Esposito, Francesco Beguinot, Mario Pirastu

**Affiliations:** Institute of Molecular Genetics-CNR, National Research Council of Italy, Via Abbiategrasso 207, 27100 Pavia, Italy; Institute of Population Genetics, National Research Council of Italy, Sassari, Italy; Unit of Human Nutrition, Department of Public Health, Experimental and Forensic Medicine, University of Pavia, Pavia, Italy; Ogliastra Genetic Park, Perdasdefogu, Italy; Dipartimento di Scienze Mediche Traslazionali, Università degli Studi di Napoli “Federico II”, Naples, Italy; Istituto di Endocrinologia ed Oncologia Sperimentale (IEOS-CNR), Naples, Italy

**Keywords:** Metabolic syndrome, Prevalence, Environmental, Genetic, Component

## Abstract

The metabolic syndrome (MetS) is a large-scale and expanding public-health and clinical threat worldwide. We investigated the determinants of MetS, assessed its prevalence and components and, estimated their genetic contribution, taking advantage of the special characteristics of Sardinian isolated populations. Inhabitants of 10 villages in Ogliastra region participated in a cross-sectional survey in 2002–2008 (n = 9,647). Blood samples, blood pressure (BP), anthropometry and, data from a standardized interview were collected. Prevalence of MetS was estimated by the direct method of standardization. Variables associated with the MetS were identified using multilevel logistic regression. Heritability was determined using variance component models. MetS Prevalence was 19.6% (95% CI 18.9–20.4%) according to NCEP-ATPIII, 24.8% (95% CI 24.0–25.6%) according to IDF and, 29% (95% CI 28.1–29.8%) according to AHA/NHLBI harmonized criteria, ranging from 9 to 26% among villages. The most prevalent combination was BP + HDL-cholesterol (HDL) + triglycerides (TRIG) (19%), followed by BP + HDL + waist circumference (WAIST) (17%) and, BP + HDL + TRIG + WAIST (13.6%). Heritability of MetS was 48% (*p* = 1.62 × 10^−25^), as the two most common combinations (BP + HDL + TRIG and BP + HDL + WAIST) showed heritability of 53 and 52%, respectively. The larger genetic components of the two most frequent combinations determining MetS deserve greater investigation in order to understand the underlying mechanisms. Besides, further studies are warranted to confirm these findings both in isolated and outbred populations.

**Electronic supplementary material:**

The online version of this article (doi:10.1186/s40064-015-1049-9) contains supplementary material, which is available to authorized users.

## Background

Metabolic Syndrome (MetS) is a metabolic condition characterized by a number of related disorders, such as abdominal obesity, glucose intolerance, disturbed plasma lipids and high blood pressure. MetS raises the risk of developing cardiovascular diseases and type 2 diabetes and represents a major health problem increasing morbidity and mortality (Kaur 2014).

MetS definition appeared for the first time about 25 years ago when this risk factors clustering and its association with insulin resistance suggested the investigators the existence of a unique pathophysiological condition (Meigs and Tracy 2000). In order to provide uniformity in the description of this phenomenon, different diagnostic criteria have been proposed for MetS. Firstly defined by The World Health Organization in 1998 (Alberti and Zimmet 1998), many international agencies and organizations subsequently proposed various definitions, among which the most widely used are: the Third Report of the National Cholesterol Education Program Expert Panel on Detection, Evaluation and Treatment of High Blood Cholesterol in Adults (NCEP-ATPIII) (National Cholesterol Education Program 2002), the International Diabetes Federation (IDF) (International Diabetes Federation 2006), and the harmonizing criteria of the International Diabetes Federation and American Heart Association/National Heart, Lung and Blood Institute (AHA/NHLBI) (Alberti et al. 2009).

MetS likely originates from a complex interaction between genetic, metabolic and environmental factors. Understanding what factors are predictive of MetS and how these factors are distributed and interrelated within different populations is important for identifying populations at risk and supporting public health interventions. Many investigators have evaluated the prevalence of MetS over time. Previous epidemiologic studies in American, Asian and, European populations have documented a higher prevalence of MetS in men, elderly, overweight/obese and physically inactive individuals, lower social classes, smokers and certain ethnic groups (Desroches and Lamarche 2007; Kolovou et al. 2007; Ervin 2009; Kumbasar et al. 2013).

In Italy, studies in the general population reported a MetS prevalence (NECP-ATPIII criteria) of 17.8% (Bonora et al. 2003) and, of 18% in women and 15% in men (Miccoli et al. 2005).

Studies carried out in Spain revealed that MetS prevalence was 28.5% (IDF), 24.8% (NECP-ATPIII) and 31% (harmonized definition) (Buckland et al. 2008; Fernández-Bergés et al. 2012).

A study conducted in a Dutch genetic isolate found a MetS prevalence of 36.8 vs 31% (IDF) and, 26.7 vs 22.8% (NECP-ATPIII), in men and women (Henneman et al. 2008). Data on two populations in the southwest of Germany showed a higher prevalence of MetS in rural (20–25%) as compared to urban (10–15%) populations (Boehm et al. 2005).

Others reported the prevalence of MetS in different ethnic groups. Lorenzo et al. compared the expression of the metabolic syndrome (NECP-ATPIII) in Spain (25.8%) and San Antonio, TX (28%), two populations with major differences regarding their cardiovascular risk profile (Lorenzo et al. 2003). Florez et al. evaluated Hispanic people from Zulia State, Venezuela and found that MetS prevalence (NCEP-ATPIII) was 31.2% and it was lower in Amerindian (17%) compared to Black (27.2%), White (33.3%) and Mixed (37.4%) (Florez et al. 2005). Some studies estimate the current prevalence of this syndrome in the United States to be up to 34% (Ervin 2009), and among native Japanese to be 41% in men and 51% in women (Oda et al. 2007). In northern India MetS prevalence was estimated as 47.5% (IDF) and 38.5% (NCEP-ATPIII) (Mangat et al. 2010).

The reasons for these ethnic disparities in MetS prevalence are not clear. Besides variations in environmental factors, an increased genetic susceptibility could explain the observed differences. The clustering of risk factors in the MetS may reflect multiple interrelations among these phenotypes and/or a manifestation of a dominant underlying common factor.

Since both genetic and environmental factors are involved in the MetS, we studied this complex phenotype in an isolated population from Sardinia (Ogliastra, Italy). Genetic isolates like Ogliastra represent an important and powerful tool in investigating genetic and non–genetic risk factors of complex diseases because of a reduced background variability.

Aims of this study are to investigate the determinants of MetS, to assess its prevalence and components, and to estimate their genetic contribution.

## Methods

### Population features and study design

Ogliastra is a mountainous region flanking the eastern coastal areas of Sardinia (Additional file 1: Figure S1). It is inhabited by small communities characterized, on the one hand, by similar environment, life style, social customs and eating habits and, on the other hand, by very few exchanges among each other because of the morphology of their territory, the distance from big towns and the inadequacy of transport links (Angius et al. 2001). Furthermore, various analyses of the Y chromosome, mitochondrial DNA and genome wide high density SNPs revealed a great deal of genetic differentiation among subpopulations within Ogliastra (Fraumene et al. 2003, 2006; Pistis et al. 2009).

A cross-sectional survey was carried out in 10 villages from this area between 2002 and 2008: Baunei, Escalaplano, Loceri, Perdasdefogu, Seui, Seulo, Talana, Triei, Urzulei and Ussassai. People living in the villages were invited to take part in the study by means of public proclamations and letters sent to each family. Respondents (average participation rate 80%) gave a blood sample, underwent anthropometric measurements, bioelectrical impedance analysis and a standardized interview collecting socio-demographic, lifestyle, medical and drug history data.

The sample analysed in the present study aged 18–101 years old and consisted of 9,647 individuals (4,075 men and 5,572 women).

### Data collection and measurements

Among living habits, we gathered information on physical activity (never/occasional/moderate/intense), current smoking (number of cigarettes smoked per day) and alcohol consumption (number of glasses per day of wine, beer and spirits). We collected data on the use of lipid-lowering drugs, antihypertensive and hypoglycemic treatments.

Blood tests (24 biochemical and 22 hemogram parameters) were made in our central laboratory in Perdasdefogu (Targa 3000, Biotecnica Instruments, Rome, Italy and Coulter LH Hematology analyzer, Beckman-Coulter, Brea, CA).

Anthropometric measurements (weight, height, waist, wrist and hip circumferences) were taken on subjects wearing only their underwear. Weight was determined on a portable electronic scale to the nearest 0.1 kg and height was measured to the nearest 0.5 cm with a stadiometer. Body mass index (BMI) in kg/m^2^ and waist hip ratio were calculated. Bioelectrical impedance analysis was performed by BIA 101 (RjL/Akern Systems, Detroit, MI): resistance, reactance and phase angle were used to determine body composition parameters. Blood pressure (BP) was measured in both arms with a standard mercury sphygmomanometer (Miniatur 300 B, Speidel & Keller) according to standard conditions. Three measurements were performed at intervals of 2–5 min and the mean of the three values was calculated considering the arm with the higher pressure.

### Metabolic syndrome

In this study NCEP-ATPIII, IDF and, AHA/NHLBI harmonized criteria defined MetS (National Cholesterol Education Program 2002; International Diabetes Federation 2006; Alberti et al. 2009). When using NCEP-ATPIII definition, subjects who had three or more of the following criteria were identified as affected by MetS: triglycerides (TRIG) ≥150 mg/dL or lipid-lowering medication; HDL-cholesterol <40 mg/dL for men and <50 mg/dL for women or lipid-lowering medication; systolic BP ≥130 mmHg or diastolic BP ≥85 mmHg or antihypertensive medication; fasting glucose (GLU) ≥110 mg/dL or hypoglycaemic medication; waist circumference >102 cm for men and >88 cm for women.

When using IDF definition, a subject was considered affected by MetS if central obesity (defined as waist circumference ≥94 cm for men and ≥80 cm for women) plus two of the following factors subsisted: TRIG ≥150 mg/dL or lipid-lowering medication; HDL-cholesterol <40 mg/dL for men and <50 mg/dL for women or lipid-lowering medication; systolic BP ≥130 mmHg or diastolic BP ≥85 mmHg or antihypertensive medication; GLU ≥100 mg/dL or previously diagnosed type 2 diabetes. Finally, when using AHA/NHLBI harmonized criteria, MetS is defined as the presence of three or more of the IDF definition’s metabolic factors.

### Statistical analysis

Statistical analysis was performed using STATA 11.1 (College Station, TX). Collected data were described by sex, using *t* test to compare mean values and Chi square test to assess homogeneity of proportions. Estimates of prevalence were standardized by the direct method using the 2008 population structure of Italy. We run ANOVA on the quantitative variables, adjusting for age and sex, for assessing discrepancies across villages. We computed the frequencies of MetS components’ combinations and evaluated variation by age, sex and village, using multinomial logistic regression. Furthermore, logistic regression was run to identify variables associated with the MetS. Given the special characteristics of genetic isolates, where individuals are nested within families and within villages, we used multilevel models (Generalized Linear Mixed Models) (Rabe-Hesketh et al. 2005), that allow family and village parameters to vary randomly. Independent quantitative variables were categorized into sex-specific quintiles to better assess the direction and strength of the association. Intraclass correlation coefficient, that provides a quantitative measure of within-cluster correlation or the proportion of the total variance in the outcome attributable to village and family effects, has been computed (Snijders and Bosker 1999). Finally, with the aim of investigating the genetic contribution to MetS, heritability analysis was carried out using SOLAR (Sequential Oligogenic Linkage Analysis Routines version 4.2.7) (Almasy and Blangero 1998), that implements variance component models on extended pedigrees. Heritability, which is the proportion of phenotypic variance attributable to additive genetic effects, was estimated for MetS and its components’ combinations as dichotomous traits and, for MetS individual components as quantitative traits, after accounting for covariates whose effect was significant at the *p* < 0.05 level. To handle discrete traits SOLAR uses a liability threshold model, that extends polygenic theory to discrete non-mendelian characters by postulating an underlying continuously variable susceptibility (Falconer and Mackay 1996). All analyses were adjusted for age, sex, treatment, smoking, alcohol and exercise. The significance of heritability estimates was tested by comparing the likelihoods of nested models using the likelihood ratio test.

## Results

A total of 9,647 individuals ranging from 18 to 101 years of age were included in the analysis. The general characteristic of the study participants are presented in Table 1. Men had higher BMI, systolic and diastolic BP, TRIG, GLU, serum uric acid, liver enzymes, white and red blood cell counts and, estimated glomerular filtration rate (eGFR), whereas women had higher total and HDL-cholesterol. In addition, the proportion of men who smoked, drank red wine and were physically active was higher than that of women. Overall, 16.3% of the study sample was obese, 38.7% had hypertension, 6.8% diabetes and 22.9% chronic kidney disease.

**Table 1:** Characteristics of the study participants

	Men (N = 4,075)	Women (N = 5,572)	*p* value*
Age (years)	49 (17)	49 (17)	0.7303
Body mass index (kg/m^2^)	26.5 (3.9)	25.3 (5.0)	<0.0001
Systolic blood pressure (mmHg)	131 (16)	125 (18)	<0.0001
Diastolic blood pressure (mmHg)	83 (10)	79 (10)	<0.0001
Antihypertensive drug use	715 (17.6%)	1,002 (18.0%)	0.580
Total cholesterol (mg/dL)	203 (40)	207 (38)	<0.0001
Triglycerides (mg/dL)	124 (98)	95 (54)	<0.0001
HDL-cholesterol (mg/dL)	49 (12)	58 (13)	<0.0001
LDL-cholesterol (mg/dL)	130 (36)	130 (34)	0.9400
Antilipidemic drug use	202 (5.0%)	306 (5.5%)	0.245
Fasting blood glucose (mg/dL)	100 (25)	93 (24)	<0.0001
Serum uric acid (mg/dL)	5.3 (1.3)	3.6 (1.1)	<0.0001
AST (U/L)	24 (16)	20 (11)	<0.0001
ALT (U/L)	32 (25)	21 (16)	<0.0001
White blood cells (×10^3^/μL)	7.4 (1.8)	6.9 (1.8)	<0.0001
Red blood cells (×10^3^/μL)	5.2 (0.6)	4.7 (0.5)	<0.0001
eGFR^a^ (mL/min/1.73 m^2^)	73 (15)	69 (16)	<0.0001
Current smokers	962 (23.6%)	728 (13.1%)	<0.0001
Wine drinkers	2,343 (57.6%)	1,302 (23.4%)	<0.0001
Phisically active	585 (14.4%)	915 (9.2%)	<0.0001

Data are presented as absolute and relative frequencies, no. (%), or means (SD).

*ALT* alanine transaminase, *AST* aspartate transaminase, *eGFR* estimated glomerular filtration rate.

^a^eGFR was calculated using the MDRD formula following the recommendations of the National Kidney Disease Education Program.

* *p* value refers to *t* test for difference in mean values, for quantitative variables, and to Chi square test for homogeneity of proportions, for categorical variables, between men and women.

**Figure 1:**
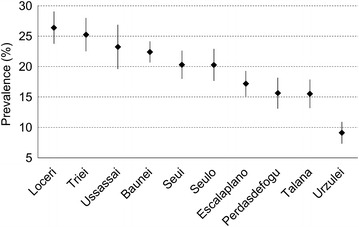
Prevalence of the metabolic syndrome by village according to NCEP-ATPIII. *Bars* are 95% CI.

Standardized prevalence of MetS was 19.6% (NCEP-ATPIII, 95% CI 18.9–20.4%), 24.8% (IDF, 95% CI 24.0–25.6%) and, 29% (IDF and AHA/NHLBI, 95% CI 28.1–29.8%), with a statistically significant difference between men and women (31.8 vs 26.4%). Since the ten villages represent genetic isolates with peculiar founder populations and almost no migration or exchanges amongst them for many centuries up to 30 years ago, prevalence of MetS was estimated separately (NCEP-ATPIII, Figure 1). In some of the villages age- and sex-adjusted prevalence was significantly higher or lower than the average value, ranging from 9 to 26%.

Aiming at explaining such differences, we evaluated all the variables we collected for studying the MetS (Additional file 2: Figure S2). Villages with highest or lowest prevalence of MetS, besides factors directly involved in its definition, have also consistent values of the variables we found associated with MetS even if in a different way: for example, Loceri (26% MetS prevalence) has also higher BMI, waist and wrist circumference, but moderate values of serum lipids, as Talana (15% MetS prevalence) has lower blood glucose and fat mass, but higher BMI and BP.

**Figure 2:**
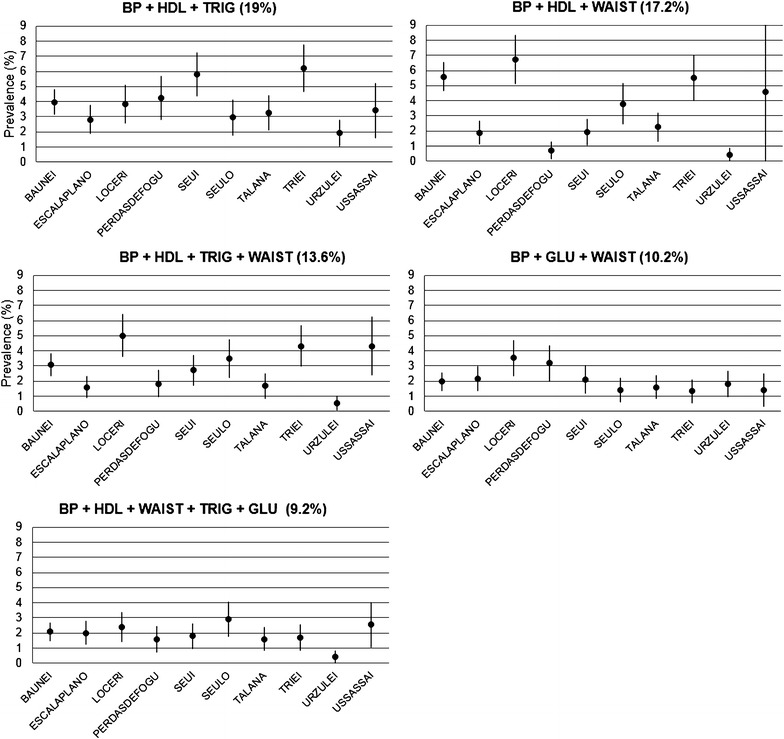
Prevalence of MetS components’ combinations by village. *Bars* are 95% CI. *Percentages in brackets* represent prevalence of specific combinations over subjects affected by MetS. Prevalences on *y* axis are computed over the entire sample adjusting for age and sex.

We investigated which components combination contributed most to the diagnosis of MetS. Table 2 shows the distribution of affected subjects by all possible combinations of components. The most prevalent combination is BP + HDL + TRIG (19%), characterizing about one-third of men. The second one is BP + HDL + WAIST (17%), representing about one-fourth of women, and the third one is BP + HDL + TRIG + WAIST (13.6%). For some of the combinations there is a statistically significant difference between men and women, in particular MetS in women appears more influenced by the waist component as MetS in men looks more influenced by the triglyceridemia and glycaemia components. Interestingly, for the most frequent combinations, statistically significant differences were observed among villages, even after adjusting for age and sex: in villages where the BP + HDL + TRIG combination was the most prevalent, the BP + HDL + WAIST was the less prevalent (Figure 2) and vice versa.

**Table 2:** Distribution of affected subjects by MetS components composition

	Men (N = 838)		Women (N = 1,121)		All (N = 1,959)	
	N	%	N	%	N	%
BP + HDL + TRIG*	241	28.76	132	11.78	373	19.04
BP + HDL + GLU*	44	5.25	18	1.61	62	3.16
BP + HDL + WAIST*	68	8.11	269	24	337	17.2
BP + TRIG + GLU	53	6.32	10	0.89	63	3.22
BP + TRIG + WAIST	52	6.21	62	5.53	114	5.82
BP + GLU + WAIST	79	9.43	121	10.79	200	10.21
HDL + TRIG + GLU	15	1.79	7	0.62	22	1.12
HDL + TRIG + WAIST*	10	1.19	48	4.28	58	2.96
HDL + GLU + WAIST	1	0.12	14	1.25	15	0.77
TRIG + GLU + WAIST	2	0.24	3	0.27	5	0.26
BP + HDL + TRIG + GLU*	72	8.59	26	2.32	98	5
BP + HDL + TRIG + WAIST*	76	9.07	190	16.95	266	13.58
BP + HDL + GLU + WAIST	24	2.86	77	6.87	101	5.16
BP + TRIG + GLU + WAIST	30	3.58	20	1.78	50	2.55
HDL + TRIG + GLU + WAIST	7	0.84	7	0.62	14	0.71
BP + HDL + WAIST + TRIG + GLU	64	7.64	117	10.44	181	9.24

*BP* blood pressure, *GLU* fasting plasma glucose, *HDL* high density lipoprotein cholesterol, *TRIG* triglycerides, *WAIST* waist circumference.

* *p* < 0.05 for difference of affected subjects’ proportions in men and women, two sided Chi square test.

In the multilevel age-adjusted logistic regression models, among anthropometric and body composition variables, upper quintiles of BMI, wrist and waist circumference, fat mass, body cell mass and intracellular water percentages, on the one hand, and lower quintiles of fat free mass, muscular mass, total body water and extracellular water percentages, on the other hand, were associated to MetS. Among serum and blood parameters, higher levels of triglycerides, blood glucose, uric acid, alanine transaminases, white and red blood cells and, lower levels of HDL-cholesterol and eGFR were associated to MetS. In addition, smoking, wine drinking, physical inactivity, young age at menarche or at least two pregnancies in women were associated with the disorder. Notably, over 55 years of age, women had about three times the odds of MetS in respect to men (Additional file 3: Table S1). Even in the multilevel multiple model, many of these variables were confirmed as independently associated to MetS (Table 3).

**Table 3:** Multiple logistic regression: independent associates of MetS

	Men^c^				Women^d^			
	Quintiles	OR^a^	95% CI	*p* value	Quintiles	OR^a^	95% CI	*p* value
Wrist (cm)	(17–17.3)	1.4	0.9, 2.1	0.089	(15–15.6)	1.6	1.1, 2.3	0.014
	(17.3–17.8)	1.1	0.8, 1.7	0.486	(15.6–16)	1.8	1.3, 2.6	0.001
	(17.9–18.3)	1.8	1.2, 2.6	0.003	(16–16.7)	2.1	1.5, 3.1	<0.0001
	(18.3–23.5)	3.1	2.2, 4.4	<0.0001	(16.7–21)	3.2	2.2, 4.5	<0.0001
Fat mass (%)	(0.15–0.19)	1.7	1.1, 2.7	0.02	(0.22–0.27)	2.5	1.4, 4.3	0.001
	(0.19–0.23)	2.6	1.7, 4	<0.0001	(0.27–0.32)	4.9	2.9, 8.2	<0.0001
	(0.23–0.27)	3	1.9, 4.7	<0.0001	(0.32–0.38)	8.8	5.3, 14.8	<0.0001
	(0.27–0.83)	5.5	3.5, 8.8	<0.0001	(0.38–0.79)	11.8	7, 20	<0.0001
Intracellular water (%)	(0.54–0.57)	1.2	0.9, 1.7	0.21	(0.51–0.53)	1.4	1.1, 1.9	0.009
	(0.57–0.58)	1.5	1, 2.2	0.03	(0.53–0.55)	1.5	1.1, 2	0.004
	(0.59–0.6)	1.7	1.1, 2.5	0.008	(0.55–0.57)	1.7	1.3, 2.3	0.001
	(0.6–0.67)	1.4	0.9, 2.1	0.162	(0.57–0.71)	2.3	1.7, 3.2	<0.0001
WBC (×10^3^/μL)	(5.9–6.7)	1	0.7, 1.4	0.968	(5.5–6.3)	1.1	0.8, 1.4	0.622
	(6.7–7.6)	1.5	1, 2.1	0.03	(6.3–7.1)	1.2	0.9, 1.6	0.123
	(7.6–8.7)	1.6	1.1, 2.4	0.007	(7.1–8.1)	1.3	1, 1.7	0.099
	(8.7–24.4)	2.4	1.7, 3.5	<0.0001	(8.2–40.7)	2	1.5, 2.7	<0.0001
RBC (×10^3^/μL)	(4.7–5)	1.1	0.8, 1.5	0.558	(4.3–4.6)	1.2	0.9, 1.6	0.26
	(5–5.3)	1.2	0.9, 1.7	0.259	(4.6–4.8)	1.2	0.9, 1.5	0.323
	(5.3–5.6)	0.9	0.6, 1.2	0.394	(4.8–5.1)	1.3	1, 1.8	0.052
	(5.6–7.8)	1.2	0.9, 1.7	0.228	(5.2–7)	1.3	1, 1.8	0.046
Hyperuricaemia^b^	UA >6 or 7 mg/dl	1.6	1.2, 2.3	0.002	UA >6 or 7 mg/dl	3.5	2.2, 5.6	<0.0001
Liver enzymes alteration	AST or ALT >40 U/L	1.8	1.4, 2.3	<0.0001	AST or ALT >40 U/L	1.7	1.3, 2.4	0.001
Cronic kidney disease	eGFR <60 ml/min/1.73m2	1.3	1, 1.7	0.058	eGFR <60 ml/min/1.73m2	1.3	1.1, 1.6	0.006
Physical activity	seldom	0.7	0.5, 1.1	0.118	Menarch age (years)			
	1–2 times/week	0.5	0.2, 0.9	0.014	≤12	1.4	1, 1.8	0.048
	>2 times/week	0.4	0.2, 0.8	0.009	Pregnancies (no)			
Smoking	Ex smoker	1.1	0.9, 1.5	0.275	≥2	1.2	1.1, 1.5	0.047
	Smoker	1.4	1, 1.9	0.041				

*CI* confidence interval, *eGFR* estimated glomerular filtration rate, *OR* odds ratio, *RBC* red blood cells, *WBC* white blood cells.

^a^Estimates are adjusted for age, wine consumption, physical activity and smoking. Reference category is the first quintile or normal range for quantitative variables and never/otherwise for qualitative variables.

^b^Serum uric acid >7.0 mg/dL in men and >6.0 mg/dL in women.

^c^Intraclass correlation coefficient (the proportion of the total variance in the outcome attributable to village and family effect) at the village level and at the family-within-village level are both ρ = 0.01811053.

^d^Intraclass correlation coefficient is ρ = 0.04958685 at the village level and ρ = 0.07525541 at the family-within-village level.

Results of heritability analysis are presented in Table 4. MetS heritability was 48% (*p* = 1.62 × 10^−25^), with no significant difference among villages. Most common combinations showed heritabilities of 53 and 52%, for BP + HDL + TRIG and BP + HDL + WAIST, respectively (Additional file 4: Figure S3, shows two pedigrees as an example of such high degree of familial aggregation); for BP + HDL + TRIG + WAIST combination heritability was 34%, and for BP + HDL + WAIST + TRIG + GLU combination it was 58%; while for BP + GLU + WAIST combination heritability (17%) was not significantly different from zero. Finally, heritability of each single MetS component was estimated, showing HDL-cholesterol and waist circumference as highly heritable traits.

**Table 4:** Heritability of MetS

Trait	Heritability (%)	*p* value*	Covariates^a^ (%)
MetS^b^	48	1.62 × 10^−25^	24
BP + HDL + TRIG	53	5.80 × 10^−9^	21
BP + HDL + WAIST	52	2.00 × 10^−7^	10
BP + HDL + TRIG + WAIST	34	0.003	19
BP + GLU + WAIST	17	0.09	14
BP + HDL + WAIST + TRIG + GLU	58	0.001	28
SBP (mmHg)	29	2.04 × 10^−53^	27
DBP (mmHg)	20	2.35 × 10^−31^	9
HDL (mg/dL)	60	7.72 × 10^−221^	10
TRIG^c^ (mg/dL)	35	4.28 × 10^−72^	6
WAIST (cm)	41	1.74 × 10^−70^	29
GLU^d^ (mg/dL)	31	2.72 × 10^−58^	38

The 9,647 individuals in this study sample were included into 589 pedigrees counting 16,463 members (mean size, 27.9 subjects).

*BP* blood pressure, *DBP* diastolic blood pressure, *GLU* fasting plasma glucose, *HDL* high density lipoprotein cholesterol, *SBP* systolic blood pressure, *TRIG* triglycerides, *WAIST* waist circumference.

^a^For quantitative traits it represents the percentage of variance explained by covariates, for dichotomous trait it represents Kullback–Leibler R-squared.

^b^MetS as a dichotomous trait, according to the NCEP-ATPIII definition.

^c^Logarithmic transformation has been applied.

^d^ reciprocal transformation has been applied.

* Two sided *p* value for testing the null hypothesis that heritability = 0.

## Discussion

Assessing prevalence of MetS and investigating what factors are associated and how these factors are distributed and interrelated in populations characterized by a great deal of environmental homogeneity, may help dissecting such a complex disease.

Although the prevalence may vary depending on geographic location, race, gender and, urbanization, MetS affects over 20% of adults in many populations worldwide. In Sardinian genetic isolates we obtained results in accordance with this worldwide trend. We found higher prevalence of MetS according to the AHA/NHLBI and IDF definitions compared to the NCEP-ATPIII one (29, 24.8 vs 19.6%) (Fernández-Bergés et al. 2012; Mangat et al. 2010). More controversial is the difference in prevalence between men and women: some studies found a higher prevalence in men (Buckland et al. 2008), some others in women (Henneman et al. 2008; Lorenzo et al. 2003) and some studies did not observe any significant difference (Bonora et al. 2003). Notably, age- and sex-adjusted prevalence of MetS varied widely among the 10 villages (9–26%). These discrepancies across villages, together with the differences/similarities observed among collected data (Additional file 2: Figure S2), were such that resembled the clustering already outlined in a previous study made on the basis of the population’s genetic structure (Pistis et al. 2009). This observation supports the existence of genetic effects that contribute to phenotypic variation among sub-populations in the same region.

Such variability prompted us to investigate the distribution of the combinations of MetS components among villages. Interestingly, for the most frequent combinations, some statistically significant differences were observed, even after adjusting for age and sex. For instance, in Perdasdefogu and Seui, BP + HDL + TRIG combination was significantly more frequent than the BP + HDL + WAIST one and vice versa in Loceri. Once sex, as a potential confounding factor, is ruled out, and given that in these populations there is a relatively homogeneous environment (Angius et al. 2001), a possible explanation may be the presence of different genetic factors in villages behaving in opposite manners. This hypothesis is supported by the high heritability showed by both these combinations (53 and 52%, respectively) and by the different genetic structure of these villages (Pistis et al. 2009). Distinctive founder effects and genetic drift may have induced a striking differentiation among villages, so that different frequencies of the allelic variants involved in the expression of these combinations may be present in these villages.

Overall, BP and HDL-cholesterol seem to be the most important components that, added to high TRIG or glycaemia in men and to large waist circumference in women, most frequently determine MetS. Furthermore, the combination of these factors is set in a condition where lifestyle, according to changes that typically occur in Western society, such as everyday physical activity decrease and improper eating habits, most influences the family predisposition to develop insulin resistance and MetS. The observed gender-specific differences confirm results of other studies (Miccoli et al. 2005; Lorenzo et al. 2003).

Logistic regression analysis on anthropometric and body composition variables confirmed that all forms of excessive body fat are associated to metabolic syndrome, as opposed to higher proportions of muscular and fat free mass.

Even adjusting for age, smoking and drinking we observed a significant association of white blood cells (WBC) and red blood cells (RBC) counts with MetS. It is well known that WBC count is a marker of acute infection, tissue damage, and other inflammatory conditions, but the mechanisms underlying its increase in MetS patients remain unclear. One possibility may be via the association between insulin resistance and WBC; another one, via proinflammatory cytokines, like TNF-α and IL-6 released from adipose cells, that elevate the WBC count (Nagasawa et al. 2004). Recent longitudinal studies suggested that baseline inflammation mirrored by WBC level may impact future MetS development (Babio et al. 2013; Kim et al. 2010). In addition, the increase in RBC count could be expression of an insulin-resistant state, since it is suggested that insulin can bind receptors upon erythropoietic cells and stimulate their proliferation (Aoki et al. 1994). Just a few studies addressed the possible association of RBC with MetS (Kim et al. 2010; Straface et al. 2011).

The observation that the prevalence of MetS increases with increasing levels of serum uric acid and, independently, with decreasing levels of e-GFR, is confirmed by other studies (Sui et al. 2008; Thomas et al. 2011). Recently, growing evidence demonstrated that uric acid may have a key role in the pathogenesis of MetS. Proposed mechanisms of uric acid mediated MetS include the inhibition of endothelial NO causing hypertension, inflammation and oxidative stress in adipocytes leading to insulin resistance and, increased endothelial and smooth muscle oxidative stress (Wang et al. 2012).

Previous studies have shown that higher concentrations of liver enzymes are associated with the diagnosis of MetS and its components and are well known steatosis markers (Kotronen et al. 2007).

Finally, our results confirm that young age at menarche might play a role in the development of the MetS, even if the pathway is not yet clear. It is possible that early menarche is a marker for childhood obesity; whether it acts additionally or as a risk factor by itself rather than through sex hormone differences is still to be understood (Stöckl et al. 2011; Glueck et al. 2013).

It is noteworthy that, overall, the analysis of quantitative parameters such as anthropometric ones, alanine aminotransferase, eGFR, white and red blood cell count or, serum uric acid highlighted that even minute changes, still within the so called normal range, could point towards a potential dysmetabolic state. Therefore, in the era of early detection and prevention of metabolic disorders, a practical recommendation might be to follow these parameters as continuous biomarkers taking into consideration the possibility that even slight changes might be relevant.

We recognize several limitations in this study. First of all, our conclusions are mainly descriptive since they are based on a cross sectional design. Besides, no exclusion criteria were applied, thus, possible confounders could have affected the results. Nonetheless, an outstanding strength of our study is that it was conducted in a large representative sample of the Sardinian adult population using standard protocols and instruments. Furthermore, adequate training of data collectors, high response rate of participants and detailed information on medical history and lifestyle ensured a high quality of this study results, as evidenced by the fact that we were able to identify many of the well known MetS risk factors.

In summary, the two most frequent combinations for MetS diagnosis, BP + HDL + TRIG and BP + HDL + WAIST, show a sex-specific element. Furthermore, they seem to have a large genetic component that could be successfully investigated in population isolates characterized by a homogeneous environment that likely reduces the background variability otherwise present in outbred populations.

## Additional files

Additional file 1: Figure S1 Ogliastra region. Geographical location of the 10 villages participating in the epidemiologic survey Additional file 2: Figure S2 Age- and sex-adjusted (ANOVA) mean values of relevant parameters among villages (*vertical bars* represent 95% CI). Distribution of lifestyle variables among villages Additional file 3: Table S1 Variables associated with MetS Additional file 4: Figure S3 Familial aggregation. Example of Ogliastra pedigrees showing familial aggregation for the combinations BP + HDL + WAIST and BP + HDL + TRIG, respectively
