# Supplementary figures and images for: Dissecting metabolic syndrome components: data from an epidemiologic survey in a genetic isolate
Source: Springerplus. 2015 Jul 7;4:324. doi: 10.1186/s40064-015-1049-9 (PMC4493262; doi:10.1186/s40064-015-1049-9)

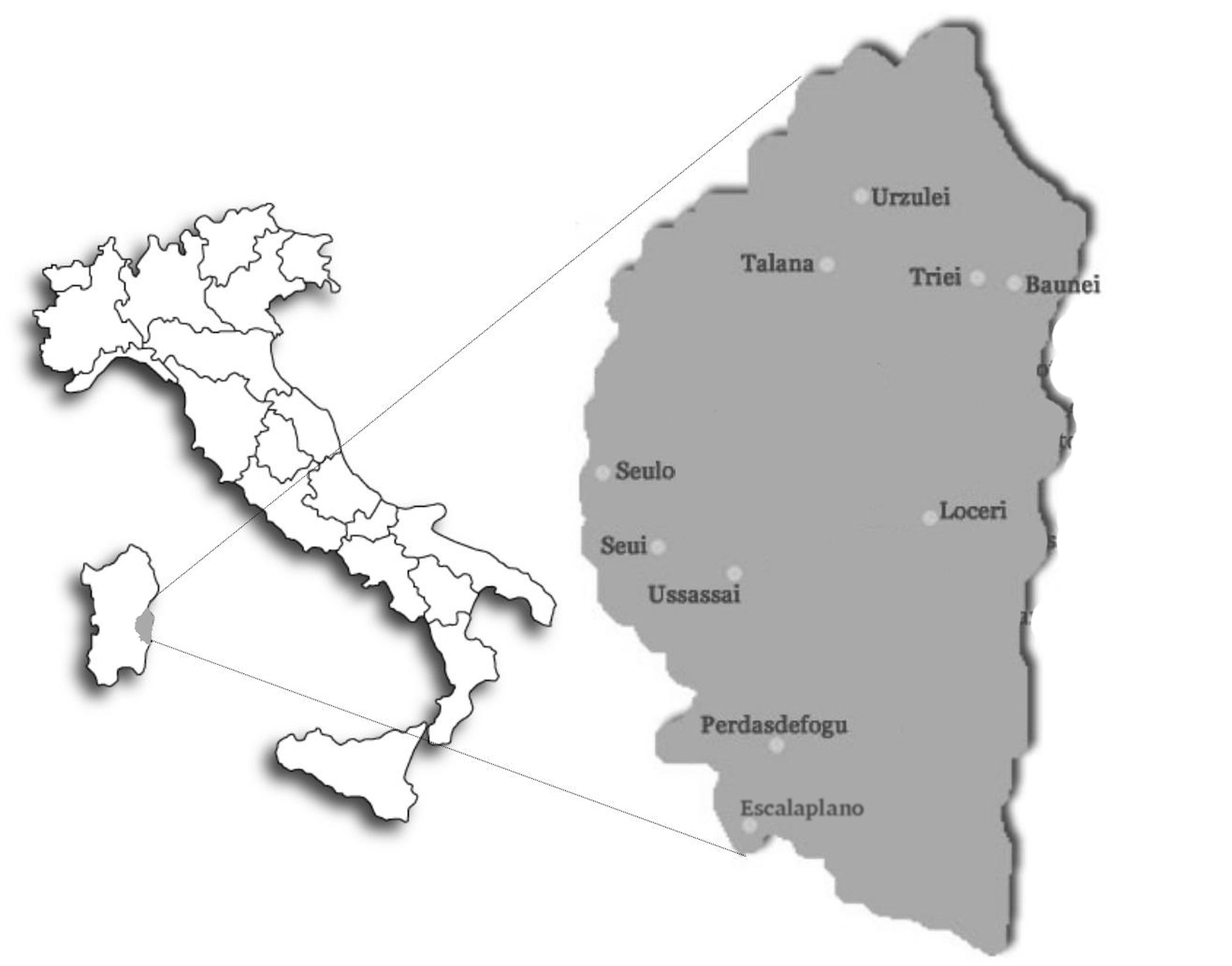

Supplement: Additional file 1: Figure S1 — Ogliastra region. Geographical location of the 10 villages participating in the epidemiologic survey [file 40064_2015_1049_MOESM1_ESM.tiff]
