# Supplementary material for: Dissecting metabolic syndrome components: data from an epidemiologic survey in a genetic isolate
Source: Springerplus. 2015 Jul 7;4:324. doi: 10.1186/s40064-015-1049-9 (PMC4493262; doi:10.1186/s40064-015-1049-9)
Supplement: Additional file 2: Figure S2 — Age- and sex-adjusted (ANOVA) mean values of relevant parameters among villages (vertical bars represent 95% CI). Distribution of lifestyle variables among villages [file 40064_2015_1049_MOESM2_ESM.docx]

DISSECTING METABOLIC SYNDROME COMPONENTS: DATA FROM AN EPIDEMIOLOGIC SURVEY IN A GENETIC ISOLATE

Acta Diabetologica

Ginevra Biino^1^, Maria Pina Concas^2^, Hellas Cena^3^, Debora Parracciani^4^, Simona Vaccargiu^2^, Massimiliano Cosso^2^, Francesca Marras^2^, Vittoria D’Esposito^6^, Francesco Beguinot^5,6^, Mario Pirastu^2^

^1^ Institute of Molecular Genetics, National Research Council of Italy, Pavia

^2^ Institute of Population Genetics, National Research Council of Italy, Sassari

^3^ Department of Public Health, Experimental and Forensic Medicine - Unit of Human Nutrition, University of Pavia, Pavia, Italy

^4^ Ogliastra Genetic Park, Perdasdefogu, Ogliastra, Italy

^5^ Istituto di Endocrinologia ed Oncologia Sperimentale (IEOS-CNR), Naples, Italy

^6^ Dipartimento di Scienze Mediche Traslazionali, Università degli Studi di Napoli “Federico II”, Naples, Italy

Corresponding author: Ginevra Biino, [biino@igm.cnr.it](mailto:biino@igm.cnr.it)

**Online Resource 2**

**Figure S2.** Age- and sex-adjusted (ANOVA) mean values of relevant parameters among villages (vertical bars represent 95% CI). Distribution of lifestyle variables among villages.
