# Supplementary material for: Dissecting metabolic syndrome components: data from an epidemiologic survey in a genetic isolate
Source: Springerplus. 2015 Jul 7;4:324. doi: 10.1186/s40064-015-1049-9 (PMC4493262; doi:10.1186/s40064-015-1049-9)
Supplement: Additional file 3: Table S1 — Variables associated with MetS [file 40064_2015_1049_MOESM3_ESM.docx]

DISSECTING METABOLIC SYNDROME COMPONENTS: DATA FROM AN EPIDEMIOLOGIC SURVEY IN A GENETIC ISOLATE

Acta Diabetologica

Ginevra Biino^1^, Maria Pina Concas^2^, Hellas Cena^3^, Debora Parracciani^4^, Simona Vaccargiu^2^, Massimiliano Cosso^2^, Francesca Marras^2^, Vittoria D’Esposito^6^, Francesco Beguinot^5,6^, Mario Pirastu^2^

^1^ Institute of Molecular Genetics, National Research Council of Italy, Pavia

^2^ Institute of Population Genetics, National Research Council of Italy, Sassari

^3^ Department of Public Health, Experimental and Forensic Medicine - Unit of Human Nutrition, University of Pavia, Pavia, Italy

^4^ Ogliastra Genetic Park, Perdasdefogu, Ogliastra, Italy

^5^ Istituto di Endocrinologia ed Oncologia Sperimentale (IEOS-CNR), Naples, Italy

^6^ Dipartimento di Scienze Mediche Traslazionali, Università degli Studi di Napoli “Federico II”, Naples, Italy

Corresponding author: Ginevra Biino, [biino@igm.cnr.it](mailto:biino@igm.cnr.it)

**Online Resource 3**

**Table S1** Variables associated with MetS.

|  | **Men^b^** | | | | |  | **Women^c^** | | | | |
| --- | --- | --- | --- | --- | --- | --- | --- | --- | --- | --- | --- |
|  | **Quintiles** | **MS (%)** | **OR^a^** | **95% CI** | ***P*-value** |  | **Quintiles** | **MS (%)** | **OR^a^** | **95% CI** | ***P*-value** |
| Age | (18-33) | 5.5 | 1.0 |  |  |  | (18-33) | 2.7 | 1.0 |  |  |
| (years) | (34-43) | 14.3 | 2.8 | 1.8, 4.1 | <0.0001 |  | (34-43) | 7.1 | 2.5 | 1.6, 4.0 | <0.0001 |
|  | (44-54) | 22.8 | 5.1 | 3.4, 7.4 | <0.0001 |  | (44-54) | 15.2 | 6.6 | 4.3, 10.1 | <0.0001 |
|  | (55-67) | 33.5 | 8.8 | 6.0, 12.8 | <0.0001 |  | (55-67) | 36.1 | 21.5 | 14.3, 32.4 | <0.0001 |
|  | (68-101) | 28.1 | 6.3 | 4.3, 9.3 | <0.0001 |  | (68-101) | 39.7 | 26.9 | 17.8, 40.5 | <0.0001 |
| Wrist | (14-16.9) | 10.9 | 1.0 |  |  |  | (12.7-15) | 6.2 | 1.0 |  |  |
| (cm) | (17-17.3) | 16.9 | 1.4 | 1.0, 1.9 | 0.079 |  | (15-15.6) | 13.6 | 1.8 | 1.2, 2.6 | 0.002 |
|  | (17.3-17.8) | 18.0 | 1.2 | 0.9, 1.8 | 0.210 |  | (15.6-16) | 19.7 | 2.5 | 1.8, 3.6 | <0.0001 |
|  | (17.9-18.3) | 25.5 | 1.9 | 1.4, 2.7 | <0.0001 |  | (16-16.7) | 27.2 | 3.2 | 2.3, 4.6 | <0.0001 |
|  | (18.3-23.5) | 37.7 | 3.7 | 2.7, 5.1 | <0.0001 |  | (16.7-21) | 42.9 | 6.0 | 4.2, 8.5 | <0.0001 |
| BMI | (13.2-23.2) | 4.3 | 1.0 |  |  |  | (14.3-20.9) | 2.4 | 1.0 |  |  |
| (kg/m^2^) | (23.2-25.3) | 11.1 | 2.2 | 1.4, 3.5 | <0.0001 |  | (20.9-23.2) | 6.1 | 2.0 | 1.2, 3.3 | 0.008 |
|  | (25.3-27.3) | 15.0 | 3.1 | 2.0, 4.8 | <0.0001 |  | (23.2-25.8) | 14.2 | 3.7 | 2.3, 5.9 | <0.0001 |
|  | (27.3-29.6) | 24.9 | 5.2 | 3.4, 7.9 | <0.0001 |  | (25.8-29.2) | 26.7 | 7.2 | 4.5, 11.5 | <0.0001 |
|  | (29.6-48.9) | 47.7 | 15.3 | 10.1, 23.1 | <0.0001 |  | (29.3-57.1) | 51.3 | 20.8 | 13.0, 33.1 | <0.0001 |
| Waist | (60.2-84.3) | 3.7 | 1.0 |  |  |  | (52.7-72.3) | 1.5 | 1.0 |  |  |
| (cm) | (84.5-90.1) | 10.2 | 2.8 | 1.7, 4.8 | <0.0001 |  | (72.4-80) | 4.0 | 1.8 | 0.9, 3.5 | 0.090 |
|  | (90.2-95.1) | 16.3 | 4.5 | 2.8, 7.5 | <0.0001 |  | (80-87) | 8.9 | 3.9 | 2.1, 7.2 | <0.0001 |
|  | (95.2-101.3) | 22.1 | 5.5 | 3.4, 9.0 | <0.0001 |  | (87.1-96) | 37.5 | 20.5 | 11.5, 36.8 | <0.0001 |
|  | (101.4-135.7) | 56.5 | 25.6 | 15.6, 42.0 | <0.0001 |  | (96.1-146.5) | 57.6 | 35.2 | 19.6, 63.5 | <0.0001 |
| Fat mass | (0-0.15) | 5.7 | 1.0 |  |  |  | (0-0.22) | 1.9 | 1.0 |  |  |
| (%) | (0.15-0.19) | 13.5 | 2.2 | 1.4, 3.3 | <0.0001 |  | (0.22-0.27) | 7.0 | 2.8 | 1.6, 4.9 | <0.0001 |
|  | (0.19-0.23) | 17.7 | 3.3 | 2.2, 4.9 | <0.0001 |  | (0.27-0.32) | 16.7 | 6.2 | 3.7, 10.5 | <0.0001 |
|  | (0.23-0.27) | 26.5 | 4.2 | 2.8, 6.3 | <0.0001 |  | (0.32-0.38) | 33.1 | 12.6 | 7.5, 21.3 | <0.0001 |
|  | (0.27-0.83) | 40.8 | 7.8 | 5.1, 11.9 | <0.0001 |  | (0.38-0.79) | 47.7 | 19.5 | 11.5, 33.3 | <0.0001 |
| Fat free mass | (0.17-0.73) | 40.2 | 1.0 |  |  |  | (0.21-0.62) | 47.4 | 1.0 |  |  |
| (%) | (0.73-0.77) | 26.3 | 0.6 | 0.4, 0.7 | <0.0001 |  | (0.62-0.68) | 32.6 | 0.6 | 0.5, 0.8 | <0.0001 |
|  | (0.77-0.81) | 17.6 | 0.4 | 0.3, 0.6 | <0.0001 |  | (0.68-0.73) | 16.9 | 0.3 | 0.3, 0.4 | <0.0001 |
|  | (0.81-0.85) | 13.7 | 0.3 | 0.2, 0.4 | <0.0001 |  | (0.73-0.78) | 6.8 | 0.1 | 0.1, 0.2 | <0.0001 |
|  | (0.85-1) | 5.4 | 0.1 | 0.1, 0.2 | <0.0001 |  | (0.78-1) | 1.8 | 0.1 | 0.0, 0.1 | <0.0001 |
| Muscular mass | (0.21-0.48) | 34.3 | 1.0 |  |  |  | (0.07-0.42) | 36.6 | 1.0 |  |  |
| (%) | (0.48-0.53) | 24.9 | 0.8 | 0.6, 1.1 | 0.141 |  | (0.42-0.47) | 27.0 | 0.9 | 0.7, 1.1 | 0.256 |
|  | (0.53-0.58) | 20.0 | 0.8 | 0.6, 1.0 | 0.050 |  | (0.47-0.51) | 17.9 | 0.5 | 0.4, 0.7 | <0.0001 |
|  | (0.58-0.63) | 13.3 | 0.5 | 0.3, 0.7 | <0.0001 |  | (0.51-0.57) | 13.3 | 0.4 | 0.3, 0.6 | <0.0001 |
|  | (0.63-1) | 10.9 | 0.4 | 0.3, 0.5 | <0.0001 |  | (0.57-1) | 10.9 | 0.4 | 0.3, 0.5 | <0.0001 |
| Body cell mass | (0.28-0.52) | 26.0 | 1.0 |  |  |  | (0.2-0.51) | 17.9 | 1.0 |  |  |
| (%) | (0.52-0.56) | 22.5 | 1.1 | 0.8, 1.5 | 0.453 |  | (0.51-0.54) | 16.3 | 1.2 | 0.9, 1.6 | 0.194 |
|  | (0.56-0.59) | 18.6 | 1.0 | 0.8, 1.4 | 0.773 |  | (0.55-0.59) | 20.2 | 1.6 | 1.2, 2.1 | 0.002 |
|  | (0.59-0.64) | 18.5 | 0.9 | 0.7, 1.3 | 0.678 |  | (0.59-0.64) | 24.8 | 1.7 | 1.2, 2.2 | 0.001 |
|  | (0.64-1) | 18.1 | 0.9 | 0.6, 1.2 | 0.343 |  | (0.64-1) | 26.8 | 1.7 | 1.3, 2.3 | <0.0001 |
| Total body water | (0.13-0.56) | 38.4 | 1.0 |  |  |  | (0.16-0.48) | 45.6 | 1.0 |  |  |
| (%) | (0.56-0.58) | 24.7 | 0.6 | 0.5, 0.8 | <0.0001 |  | (0.48-0.51) | 29.2 | 0.5 | 0.4, 0.6 | <0.0001 |
|  | (0.58-0.6) | 19.0 | 0.4 | 0.3, 0.6 | <0.0001 |  | (0.51-0.54) | 17.7 | 0.3 | 0.2, 0.4 | <0.0001 |
|  | (0.6-0.63) | 15.8 | 0.4 | 0.3, 0.5 | <0.0001 |  | (0.54-0.58) | 9.0 | 0.2 | 0.1, 0.2 | <0.0001 |
|  | (0.63-0.77) | 5.4 | 0.1 | 0.1, 0.2 | <0.0001 |  | (0.58-0.74) | 3.8 | 0.1 | 0.0, 0.1 | <0.0001 |
| Extracellular water | (0.33-0.4) | 15.9 | 1.0 |  |  |  | (0.29-0.43) | 18.1 | 1.0 |  |  |
| (%) | (0.4-0.42) | 18.6 | 1.2 | 0.8, 1.6 | 0.365 |  | (0.43-0.45) | 17.4 | 0.7 | 0.5, 1.0 | 0.028 |
|  | (0.42-0.44) | 19.8 | 1.1 | 0.8, 1.5 | 0.763 |  | (0.45-0.47) | 19.3 | 0.7 | 0.5, 0.9 | 0.007 |
|  | (0.44-0.46) | 21.2 | 0.8 | 0.6, 1.1 | 0.173 |  | (0.47-0.49) | 22.8 | 0.6 | 0.5, 0.9 | 0.003 |
|  | (0.46-0.64) | 28.4 | 0.7 | 0.5, 1.0 | 0.063 |  | (0.49-0.8) | 28.6 | 0.5 | 0.3, 0.6 | <0.0001 |
| Intracellular water | (0.36-0.54) | 28.4 | 1.0 |  |  |  | (0.2-0.51) | 28.9 | 1.0 |  |  |
| (%) | (0.54-0.57) | 20.8 | 1.1 | 0.8, 1.5 | 0.402 |  | (0.51-0.53) | 22.1 | 1.4 | 1.1, 1.8 | 0.015 |
|  | (0.57-0.58) | 19.1 | 1.5 | 1.1, 2.0 | 0.017 |  | (0.53-0.55) | 19.0 | 1.5 | 1.1, 1.9 | 0.006 |
|  | (0.59-0.6) | 19.4 | 1.7 | 1.2, 2.4 | 0.001 |  | (0.55-0.57) | 17.7 | 1.6 | 1.2, 2.2 | 0.001 |
|  | (0.6-0.67) | 15.6 | 1.4 | 1.0, 2.1 | 0.067 |  | (0.57-0.71) | 17.9 | 2.2 | 1.6, 2.9 | <0.0001 |
| Triglycerides | (21.4-68) | 5.5 | 1.0 |  |  |  | (15.7-56.4) | 4.8 | 1.0 |  |  |
| (mg/dL) | (68-87.2) | 9.7 | 1.8 | 1.1, 2.7 | 0.010 |  | (56.4-73) | 9.3 | 1.4 | 0.9, 2.1 | 0.106 |
|  | (87.2-113.5) | 14.6 | 2.7 | 1.8, 4.1 | <0.0001 |  | (73-92.6) | 15.1 | 2.3 | 1.6, 3.4 | <0.0001 |
|  | (113.5-160.4) | 20.0 | 4.8 | 3.2, 7.2 | <0.0001 |  | (92.7-124.1) | 20.5 | 3.4 | 2.4, 4.9 | <0.0001 |
|  | (160.5-2155) | 53.0 | 30.1 | 20.3, 44.6 | <0.0001 |  | (124.2-870.7) | 50.9 | 15.6 | 10.9, 22.3 | <0.0001 |
| HDL-cholesterol | (14-39) | 52.3 | 21.5 | 14.9, 31.1 | <0.0001 |  | (17.7-47.1) | 49.8 | 20.1 | 14.5, 27.7 | <0.0001 |
| (mg/dL) | (39-44.7) | 19.8 | 3.9 | 2.7, 5.6 | <0.0001 |  | (47.1-53.8) | 22.3 | 4.4 | 3.2, 6.0 | <0.0001 |
|  | (44.7-50.3) | 13.4 | 2.4 | 1.6, 3.5 | <0.0001 |  | (53.8-60) | 11.1 | 1.5 | 1.1, 2.1 | 0.021 |
|  | (50.4-58.1) | 9.3 | 1.5 | 1.0, 2.2 | 0.061 |  | (60-68.1) | 9.0 | 1.2 | 0.8, 1.7 | 0.380 |
|  | (58.1-121) | 7.5 | 1.0 |  |  |  | (68.1-140) | 8.3 | 1.0 |  |  |
| Blood glucose | (56.4-86) | 9.3 | 1.0 |  |  |  | (54.2-80.6) | 5.8 | 1.0 |  |  |
| (mg/dL) | (86-91.9) | 11.2 | 0.9 | 0.7, 1.4 | 0.776 |  | (80.6-85.6) | 9.3 | 1.3 | 0.9, 1.8 | 0.202 |
|  | (91.9-97.5) | 14.1 | 1.3 | 0.9, 1.8 | 0.121 |  | (85.7-91) | 12.6 | 1.4 | 1.0, 2.0 | 0.055 |
|  | (97.5-106.8) | 17.4 | 1.5 | 1.1, 2.1 | 0.012 |  | (91-99) | 20.8 | 2.5 | 1.8, 3.4 | <0.0001 |
|  | (106.8-436) | 50.9 | 6.9 | 5.0, 9.4 | <0.0001 |  | (99-426.9) | 52.2 | 8.1 | 5.8, 11.2 | <0.0001 |
| AST | (8.8-17.3) | 21.0 | 1.0 |  |  |  | (5.8-14.3) | 14.1 | 1.0 |  |  |
| (U/L) | (17.3-20) | 18.0 | 0.9 | 0.6, 1.1 | 0.264 |  | (14.3-16.4) | 15.4 | 0.8 | 0.6, 1.1 | 0.234 |
|  | (20-22.9) | 17.7 | 0.9 | 0.7, 1.2 | 0.575 |  | (16.4-18.7) | 20.3 | 0.8 | 0.6, 1.1 | 0.170 |
|  | (22.9-28) | 20.5 | 1.1 | 0.8, 1.4 | 0.563 |  | (18.7-22) | 20.5 | 0.8 | 0.6, 1.1 | 0.109 |
|  | (28-412) | 25.7 | 1.5 | 1.1, 1.9 | 0.005 |  | (22-209) | 30.4 | 1.0 | 0.8, 1.4 | 0.772 |
| ALT | (5.4-17.6) | 15.5 | 1.0 |  |  |  | (4.3-12) | 10.5 | 1.0 |  |  |
| (U/L) | (17.6-23) | 17.6 | 1.6 | 1.2, 2.1 | 0.003 |  | (12-15.3) | 14.0 | 1.0 | 0.8, 1.4 | 0.879 |
|  | (23-29.4) | 20.6 | 2.3 | 1.7, 3.2 | <0.0001 |  | (15.3-19) | 21.2 | 1.7 | 1.3, 2.3 | <0.0001 |
|  | (29.4-40.6) | 22.0 | 2.5 | 1.9, 3.5 | <0.0001 |  | (19-26) | 23.3 | 2.2 | 1.7, 3.0 | <0.0001 |
|  | (40.7-948) | 27.4 | 4.2 | 3.1, 5.8 | <0.0001 |  | (26.1-260.5) | 31.8 | 3.1 | 2.3, 4.2 | <0.0001 |
| Serum uric acid | (1-4.2) | 12.6 | 1.0 |  |  |  | (0.9-2.7) | 6.6 | 1.0 |  |  |
| (mg/dL) | (4.2-4.9) | 16.7 | 1.3 | 1.0, 1.8 | 0.066 |  | (2.7-3.2) | 12.8 | 2.0 | 1.4, 2.8 | <0.0001 |
|  | (4.9-5.6) | 16.5 | 1.3 | 1.0, 1.8 | 0.068 |  | (3.2-3.7) | 15.9 | 2.1 | 1.5, 2.9 | <0.0001 |
|  | (5.6-6.4) | 23.1 | 2.0 | 1.5, 2.6 | <0.0001 |  | (3.7-4.4) | 21.8 | 2.9 | 2.1, 4.0 | <0.0001 |
|  | (6.4-21) | 34.0 | 3.3 | 2.4, 4.4 | <0.0001 |  | (4.4-11.7) | 43.7 | 6.1 | 4.5, 8.3 | <0.0001 |
| eGFR | (4.6-61.3) | 33.1 | 3.0 | 2.1, 4.2 | <0.0001 |  | (7.8-56.2) | 38.9 | 2.1 | 1.5, 2.9 | <0.0001 |
| (mL/min/1.73m^2^) | (61.4-68.8) | 26.1 | 2.5 | 1.8, 3.6 | <0.0001 |  | (56.2-63.7) | 26.6 | 1.8 | 1.3, 2.5 | <0.0001 |
|  | (68.8-75.9) | 18.7 | 1.9 | 1.4, 2.7 | <0.0001 |  | (63.7-71) | 16.2 | 1.2 | 0.9, 1.7 | 0.280 |
|  | (75.9-84.3) | 15.8 | 1.8 | 1.3, 2.5 | 0.001 |  | (71-81.2) | 10.4 | 1.0 | 0.7, 1.4 | 0.977 |
|  | (84.4-146.9) | 9.1 | 1.0 |  |  |  | (81.2-154.3) | 8.5 | 1.0 |  |  |
| WBC | (1-5.9) | 16.2 | 1.0 |  |  |  | (2.3-5.5) | 17.7 | 1.0 |  |  |
| (×10^3^/μL) | (5.9-6.7) | 15.9 | 1.1 | 0.8, 1.4 | 0.713 |  | (5.5-6.3) | 19.6 | 1.4 | 1.1, 1.8 | 0.009 |
|  | (6.7-7.6) | 21.1 | 1.5 | 1.1, 2.1 | 0.004 |  | (6.3-7.1) | 19.5 | 1.7 | 1.3, 2.2 | <0.0001 |
|  | (7.6-8.7) | 22.6 | 1.8 | 1.3, 2.4 | <0.0001 |  | (7.1-8.1) | 20.2 | 1.7 | 1.3, 2.2 | <0.0001 |
|  | (8.7-24.4) | 26.9 | 2.6 | 1.9, 3.5 | <0.0001 |  | (8.2-40.7) | 23.2 | 3.0 | 2.3, 3.9 | <0.0001 |
| RBC | (2.1-4.7) | 21.0 | 1.0 |  |  |  | (3.1-4.3) | 15.7 | 1.0 |  |  |
| (×10^3^/μL) | (4.7-5) | 20.0 | 1.1 | 0.9, 1.5 | 0.330 |  | (4.3-4.6) | 18.0 | 1.2 | 0.9, 1.6 | 0.173 |
|  | (5-5.3) | 22.1 | 1.5 | 1.1, 1.9 | 0.006 |  | (4.6-4.8) | 18.3 | 1.3 | 1.0, 1.7 | 0.054 |
|  | (5.3-5.6) | 17.6 | 1.1 | 0.8, 1.4 | 0.673 |  | (4.8-5.1) | 24.4 | 1.7 | 1.3, 2.2 | <0.0001 |
|  | (5.6-7.8) | 21.9 | 1.5 | 1.1, 1.9 | 0.008 |  | (5.2-7) | 24.3 | 1.8 | 1.4, 2.4 | <0.0001 |
| Systolic blood pressure | (83-120) | 7.2 | 1.0 |  |  |  | (60-110) | 2.9 | 1.0 |  |  |
| (mmHg) | (121-125) | 11.9 | 2.0 | 1.2, 3.1 | 0.004 |  | (111-120) | 9.3 | 2.4 | 1.6, 3.6 | <0.0001 |
|  | (125-132) | 23.6 | 3.9 | 2.9, 5.4 | <0.0001 |  | (120-130) | 23.7 | 5.5 | 3.8, 8.0 | <0.0001 |
|  | (132-142) | 29.6 | 5.0 | 3.7, 6.8 | <0.0001 |  | (131-140) | 37.7 | 8.0 | 5.4, 11.7 | <0.0001 |
|  | (142-220) | 34.3 | 5.0 | 3.6, 6.8 | <0.0001 |  | (140-200) | 44.3 | 8.4 | 5.7, 12.3 | <0.0001 |
| Diastolic blood pressure | (40-75) | 10.5 | 1.0 |  |  |  | (40-70) | 6.5 | 1.0 |  |  |
| (mmHg) | (76-80) | 13.7 | 1.2 | 0.9, 1.7 | 0.195 |  | (70-77) | 12.4 | 1.5 | 1.1, 2.1 | 0.025 |
|  | (80-85) | 22.6 | 2.2 | 1.6, 3.0 | <0.0001 |  | (77-80) | 16.8 | 1.8 | 1.4, 2.5 | <0.0001 |
|  | (85-90) | 29.0 | 2.9 | 2.2, 3.9 | <0.0001 |  | (80-87) | 32.1 | 3.4 | 2.5, 4.7 | <0.0001 |
|  | (90-135) | 34.5 | 3.8 | 2.8, 5.1 | <0.0001 |  | (87-150) | 40.1 | 4.3 | 3.3, 5.7 | <0.0001 |
| Wine | never | 19.3 | 1.0 |  |  |  | never | 19.9 | 1.0 |  |  |
|  | seldom | 18.2 | 0.9 | 0.7, 1.2 | 0.566 |  | seldom | 15.7 | 0.7 | 0.6, 0.9 | 0.001 |
|  | 1-2 glass/day | 21.4 | 1.1 | 0.9, 1.4 | 0.242 |  | 1-2 glass/day | 25.2 | 1.4 | 1.2, 1.6 | <0.0001 |
|  | 0.5 litre/day | 22.7 | 1.2 | 0.9, 1.6 | 0.125 |  | 0.5 litres/day | 41.2 | 2.8 | 1.1, 7.4 | 0.036 |
|  | ≥1 litre/day | 23.7 | 1.3 | 0.9, 1.8 | 0.133 |  | ≥1 litre/day | 0.0 |  |  |  |
| Physical activity | never | 24.1 | 1.0 |  |  |  | never | 22.2 | 1.0 |  |  |
|  | seldom | 16.3 | 0.7 | 0.5, 1 | 0.043 |  | seldom | 9.9 | 0.9 | 0.6, 1.4 | 0.594 |
|  | 1 - 2 times/week | 7.8 | 0.3 | 0.2, 0.6 | <0.0001 |  | 1 - 2 times/week | 9.5 | 0.7 | 0.4, 1.1 | 0.144 |
|  | >2 times/week | 5.5 | 0.3 | 0.2, 0.5 | <0.0001 |  | >2 times/week | 7.5 | 0.6 | 0.3, 1 | 0.065 |
| Smoking | never | 17.9 | 1.0 |  |  |  | never | 22.9 | 1.0 |  |  |
|  | ex smoker | 25.5 | 1.4 | 1.1, 1.7 | 0.002 |  | ex smoker | 13.7 | 0.9 | 0.7, 1.3 | 0.608 |
|  | smoker | 17.2 | 1.4 | 1.1, 1.8 | 0.007 |  | smoker | 9.1 | 0.8 | 0.6, 1.2 | 0.293 |
| Menarch age |  |  |  |  |  |  | 8-12 | 16.2 | 1.3 | 1.2, 1.7 | 0.044 |
| (years) |  |  |  |  |  |  | 13 | 16.7 | 1.1 | 0.8, 1.4 | 0.509 |
|  |  |  |  |  |  |  | 14 | 20.6 | 1.1 | 0.9, 1.4 | 0.332 |
|  |  |  |  |  |  |  | 15 | 22.0 | 0.9 | 0.7, 1.2 | 0.400 |
|  |  |  |  |  |  |  | ≥16 | 32.4 | 1.0 |  |  |
| Pregnancies |  |  |  |  |  |  | 0 | 10.7 | 1.0 |  |  |
| (n°) |  |  |  |  |  |  | 1 | 12.3 | 1.1 | 0.8, 1.6 | 0.548 |
|  |  |  |  |  |  |  | 2 | 19.1 | 1.4 | 1.1, 1.7 | 0.012 |
|  |  |  |  |  |  |  | 3 | 27.3 | 1.6 | 1.2, 2.0 | <0.0001 |
|  |  |  |  |  |  |  | ≥4 | 38.5 | 1.6 | 1.3, 2.1 | <0.0001 |

ALT, alanine transaminase; AST, aspartate transaminase; CI, confidence interval; eGFR, estimated glomerular filtration rate; OR, odds ratio; RBC; red blood cells; WBC, white blood cells.

^a^ OR along with 95% CI and *P*-values refer to models where the tested variable is age-adjusted, except for model testing age.

^b^ intraclass correlation coefficient (the proportion of the total variance in the outcome attributable to village and family effect) ranges between ρ= 0.011758697 and ρ= 0.059953744at the village level and between ρ= 0.013606472 and ρ= 0.059953744 at the family-within-village level.

^c^ intraclass correlation coefficient ranges between ρ= 0.013145356 and ρ=0.107088765 at the village level and between ρ= 0.041088302 and ρ=0.137406728 at the family-within-village level.
